# Lipid phosphate phosphatase 3 in smooth muscle cells regulates angiotensin II-induced abdominal aortic aneurysm formation

**DOI:** 10.1038/s41598-022-08422-7

**Published:** 2022-04-05

**Authors:** Patrick M. Van Hoose, Liping Yang, Maria Kraemer, Margo Ubele, Andrew J. Morris, Susan S. Smyth

**Affiliations:** 1 Gill Heart and Vascular Institute, University of Kentucky, 741 South Limestone BBSRB, Rm: B347, Lexington, KY 40536-0509 USA; 2 Lexington Veterans Affair Medical Center, Lexington, KY USA

**Keywords:** Cardiovascular biology, Cardiology

## Abstract

Genetic variants that regulate lipid phosphate phosphatase 3 (LPP3) expression are risk factors for the development of atherosclerotic cardiovascular disease. LPP3 is dynamically upregulated in the context of vascular inflammation with particularly heightened expression in smooth muscle cells (SMC), however, the impact of LPP3 on vascular pathology is not fully understood. We investigated the role of LPP3 and lysophospholipid signaling in a well-defined model of pathologic aortic injury and observed Angiotensin II (Ang II) increases expression of *PLPP3* in SMCs through nuclear factor kappa B (NF-κB) signaling *Plpp3* global reduction (*Plpp3*^+*/−*^*)* or SMC-specific deletion (SM22-Δ) protects hyperlipidemic mice from AngII-mediated aneurysm formation. LPP3 expression regulates SMC differentiation state and lowering LPP3 levels promotes a fibroblast-like phenotype. Decreased inactivation of bioactive lysophosphatidic acid (LPA) in settings of LPP3 deficiency may underlie these phenotypes because deletion of LPA receptor 4 in mice promotes early aortic dilation and rupture in response to AngII. LPP3 expression and LPA signaling influence SMC and vessel wall responses that are important for aortic dissection and aneurysm formation. These findings could have important implications for therapeutics targeting LPA metabolism and signaling in ongoing clinical trials.

## Introduction

Lysophosphatidic acid (LPA) and sphingosine-1-phosphate (S1P) are bioactive lipids with roles in migration^1^, hematopoietic cell trafficking^2,3^, proliferation, survival and vascular development^4,5^. Endothelial cells, platelets and vascular smooth muscle cells (SMC) respond to LPA and S1P via discrete G-protein coupled receptors (LPAR 1-6, S1PR 1-5)^6^. LPA is generated by the extracellularly-secreted lysophospholipase D (lysoPLD) autotaxin (ATX) that hydrolyzes lysophosphatidylcholine (LPC)^7–10^, while S1P is generated intracellularly by sphingosine kinase 1 and 2-mediated phosphorylation of sphingosine^11^. Extracellular, biologically active lysophospholipids including LPA and S1P are inactivated by a family of lipid phosphate phosphatases (LPPs, LPP1, LPP2, and LPP3) that dephosphorylate a broad range of lipid phosphates generating receptor inactive products. LPPs contain 6 predicted transmembrane helices that are orientated with their active site on the extracellular or luminal surface with both the N- and C-terminal sequences on the cytoplasmic face^12^. Whereas LPPs dephosphorylate extracellular lipids, intracellular S1P is also degraded by S1P lyase and S1P phosphatases^6^. Mouse models of targeted inactivation of the genes encoding LPPs (*Plpp1-3*) reveal non-redundant functions for the enzymes. In particular, genetic deficiency of *Plpp3* that encodes LPP3 results in embryonic lethality in mice due to the failure of extra-embryonic vasculature formation^13^.

Evidence from genetic association studies in humans has also highlighted a unique role for LPP3 as a risk factor for the development of coronary artery disease^14–16^. Polymorphisms that disrupt C/EBPβ binding to a putative *PLPP3* enhancer region are associated with lower *PLPP3* expression and heightened risk of myocardial infarction^14–16^. These observations have focused attention on the role of LPP3 in atherosclerotic vascular disease. Loss of LPP3 in SMCs results in increased intimal hyperplasia in vascular injury^17^, perhaps in part due to heightened LPA signaling. Additionally, global reduction in *Plpp3* expression and SMC-specific inactivation of *Plpp3* promotes the development of experimental atherosclerosis in mice^18^. Together, these results indicate that LPP3, perhaps in part due to its ability to degrade LPA and thereby limit LPA signaling functions to mitigate SMC proliferation, vascular inflammation and SMC phenotypic switching. Regulation of LPP3 expression either during development or in adults with vascular disease is presently not well understood.

LPP3 is strongly upregulated during vascular inflammation^17,18^, potentially as a consequence of functional NF-kappaB (NF-κB) response elements in the promoter^19^. In exploring upstream signaling pathways that may regulate LPP3 expression, we identified angiotensin II (AngII) as a strong activator of NF-κB-dependent *PLPP3* expression in SMCs. In an attempt to understand the biological relevance of this observation, we found that global and SMC-specific loss of LPP3 expression protects hyperlipidemic mice from the development of aortic aneurysm and transmural rupture following AngII infusion. Our findings reveal a unique role for SMC LPP3 in modifying phenotypic responses that occur with vascular response to injury.

## Material and methods

The data that support the findings of this study are available from the corresponding author upon reasonable request.

### Cell culture and treatment

Human coronary artery smooth muscle cells (caHSMCs, Lonza Cat #CC-2583) were cultured in SmBM media (Lonza, Cat #CC-3181) with 5% fetal bovine serum and SmGM-2 SingleQuot Kit (Lonza, CC-4149) and maintained at 37 °C in a humidified atmosphere containing 5% CO_2_. Cells were used between passage 3–7 and at 80–90% confluency before treatment. caHSMCs were serum starved for 12 h and treated with Mβ-CD Cholesterol (10 µγ/ul, Sigma, Cat. #C4951) LPA (1 µM), AngII (1 µM, Bachem, Cat. # 4006473), transforming growth factor beta 1 (TGFβ) (1 µM, R&D Systems, Cat. #240-B002), parthenolide (1 µM, Sigma Cat. #P0667), Irbesartan (Sigma, Cat. #61188) or PD-123-319 (Sigma, Cat. #P186) for either: 24, 48 or 72 h.

### Mice

All procedures conformed to the recommendations of the Guide for the Care and Use of Laboratory Animals (Department of Health, Education, and Welfare publication number National Institutes of Health 78-23, 1996) and were approved by the University of Kentucky Institutional Animal Care and Use Committee. The study was carried out in compliance with the ARRIVE guidelines. The production and characterization of mice in which *Plpp3* is depleted in SMC by crossing *Plpp3*fl/fl mice with mice carrying Cre transgene under the SM22 promoter has been previously described^17^. The mice were backcrossed to the *Ldlr*^*−/−*^ (B6.129S7-Ldlrtm1Her/J, The Jackson Laboratory) to generate *Plpp3*fl/fl *Ldlr*^*−/−*^ (fl/fl) and *Plpp3*fl/fl SM22Cre+ *Ldlr*^*−/−*^* (*SM22-Δ) animals. Mice lacking Lpar4 have been previously described^20,21^ and were backcrossed to C57BL/6 J mice (Stock #000664, Jackson Laboratory). *Plpp3* heterozygous (*Plpp3*^+*/−*^*)* mice were generated by backcrossing *Plpp3*fl/fl mice with B6.C-Tg (CMV-Cre)1Cgn/J mice (Stock #006054, Jackson Laboratory), then *Plpp3*WT/fl Cre+ were crossed with C57BL/6 J mice (Stock #000664, Jackson Laboratory) to generate *Plpp3*^+*/−*^ mice.

### Abdominal aortic aneurysm model

Male and female SM22-Δ and fl/fl mice (on the *Ldlr*^*−/−*^ background) were placed on Western diet (Research Diets, Cat. # D12079B) at 8–12 weeks of age. One week later, subcutaneous osmotic mini pumps (Alzet, Model 2004, 0.25 µl/h) were implanted to deliver vehicle or AngII (1000 ng/kg/min, Bachem Cat. #H-1706) for 7 or 28 days. Mice received buprenorphine S.R (1.2–1.5 mg/kg) for post procedure analgesics.

Male C57BL/6J, *Plpp3*^+/−^, *LPAR4*^*Y/−*^, and their respective wild-type (WT) littermate control mice (8–12 weeks old) were injected with adeno-associated virus expressing PCSK9 (PCSK9D377Y.AAV; 20 × 10^10^ genomic copies, University of Pennsylvania Vector Core) and fed Western diet (Research Diets, Cat. # D12079B) two weeks prior to and up to 4 weeks during AngII infusion. Mice were anesthetized with inhaled isoflurane, and angiotensin II (1000 ng/kg/min, Cat. #4006473, Bachem) was infused subcutaneously via osmotic mini pump (Alzet, Model 2004, 0.25 µl/h) for 7 or 28 days. Mice were given buprenorphine S.R (1.2–1.5 mg/kg) as post procedure analgesics. Only male mice were used for these studies as female mice are resistant to Ang II-induced AAA.

### Systolic blood pressure

Systolic blood pressure was measured daily using CODA blood pressure analysis tail cuff system (Kent Scientific Corporation) after 1 week of training. Tail-cuff blood pressure measurements were assessed in the morning (10 am) and blood pressure was measured each day for at least 5 days during the duration of 4 week AngII infusion.

### Plasma cholesterol and triglyceride levels

Baseline blood samples were collected via submandibular bleed, and post AngII infusion samples were collected via retro-orbital bleed. Blood was collected in CTAD:EDTA and centrifuged at 2000x*g* for 5 min to separate plasma. Total plasma cholesterol was measured with the Wako Cholesterol E Kit (Wako Diagnostics, Cat. #999-02601) and plasma triglycerides were measured using the Wako L-Type Triglyceride M kit (Wako Diagnostics, Cat. #994-02891), according to the manufacturer’s instructions.

### Abdominal aorta ultrasound

Mice were anesthetized with inhaled isoflurane and ultrasound obtained using a 50-MHz linear probe (Vevo3100). The suprarenal abdominal aorta lumen approximately 0.5 to 2.0 mm above the right renal artery was imaged and VevoLab software version 5.5.1 (https://www.visualsonics.com/resource/vevo-lab-software) was used to measure maximal abdominal aorta luminal diameter.

### Real time (qRT) PCR and gene array

caHSMCs were lysed and collected in Trizol (ThermoFisher Scientific, Cat. #15596026) and stored at − 80 °C. Abdominal aorta tissues were collected and snap frozen. RNA was isolated using Trizol, according to manufacturer’s protocol. cDNA was generated using High-Capacity cDNA Reverse Transcription Kit (ThermoFisher Scientific, Cat. # 4368814) according to manufacturer’s protocol. qRT-PCR and custom gene array (ThermoFisher Scientific) was performed on QuantStudio 7 Flex (Applied Biosystems) using TaqMan Universal PCR Master Mix and TaqMan FAM primers with 18s as an internal control.

### Abdominal aorta histology and immunohistochemistry

Abdominal aortas were collected and embedded in OCT, and serial cross section (6 µM) obtained from the last intercostal artery until the right renal artery, which is defined as the abdominal aorta. Abdominal aorta tissue sections were stained with hematoxylin and eosin (H&E; Sigma Aldrich) and Movat Pentachrome (Poly Scientific R&D Corps, Cat. #K042) according to manufacturer’s instructions. Immunofluorescence and immunohistochemistry were performed for smooth muscle α-actin (ACTA2, Sigma Aldrich, Cat. #A5441), LPP3^22,23^, vimentin (Cell Signaling, Cat. #5741), and calponin (Abcam, Cat. # Ab46794). Immunofluorescence was quantified with Image J.

### In situ hybridization and proximity ligation assays (ISH-PLA)

Dimethylation of lysine 4 on histone 3 (H3K4me2) of the smooth muscle myosin heavy chain 11 (*Myh11*) promoter was detected by in situ hybridization (ISH) and Proximity Ligation Assay (PLA) as previously published^24^. Briefly, the 2 kb promoter of *Myh11* was amplified by PCR, cloned into pCR2.1 vector for amplification (TOPO cloning kit, ThermoFisher Scientific) and biotin labeled probes were generated by Nick Translation (Roche) using biotin-14-ATP (ThermoFisher Scientific). Probes were denatured in hybridization buffer (2X SSC, 50% high grade formamide, 10% dextran sulfate, 1 μg mouse Cot-1 DNA) for 5 min at 80 °C. Abdominal aorta sections were incubated with 0.5% pepsin at 37 °C for 15 min, followed by incubation with hybridization buffer containing biotinylated probe for 5 min at 80 °C then followed by incubation for 16–24 h at 37 °C. Following hybridization, slides were washed in 2X SSC, 0.1% NP-40 buffer. Sections were blocked, incubated with mouse H3K4dime (5 μg/mL, Millipore Sigma, clone CMA303) and rabbit Biotin (5 μg/mL, Abcam #ab53494) antibodies overnight at 4 °C, followed by incubated with secondary antibodies containing PLA probe at 37 °C for 1 h. Ligation and amplification were performed (Duolink detection kit Orange 555 nm) and mounting medium with DAPI was used. Immunofluorescence was quantified with Image J.

### Aortic smooth muscle cell isolation

Aortic smooth muscle cells were isolated from fl/fl and SM22-Δ mice as previously described^17^ and used in experiments before the first passage. Cells were serum starved for 12 h and treated with 1 µM AngII for 24 h.

### Statistical analysis

All results were expressed as mean ± SEM. All data were analyzed for normality by SharpiroWilk normality test and Brown-Forsythe equal variance prior to parametric analysis or non-parametric analysis. Data was analyzed by Student t-test or ANOVA if data was parametric and if data was non-parametric Mann–Whitney rank sum test, Chi square, or Fisher exact test was used to analyze data. Chi square analysis was used for AAA incidence. Experiments containing small size have limitations in drawing conclusions. Statistical analysis was performed using SigmaPlot software version 14 (Systat Software Inc). Box plots are shown with median (solid line) and mean (dashed line). A *P* < 0.05 was considered significant.

## Results

### AngII-increases PLPP3 expression in smooth muscle cells through an NF-κB dependent pathway

**Figure 1:**
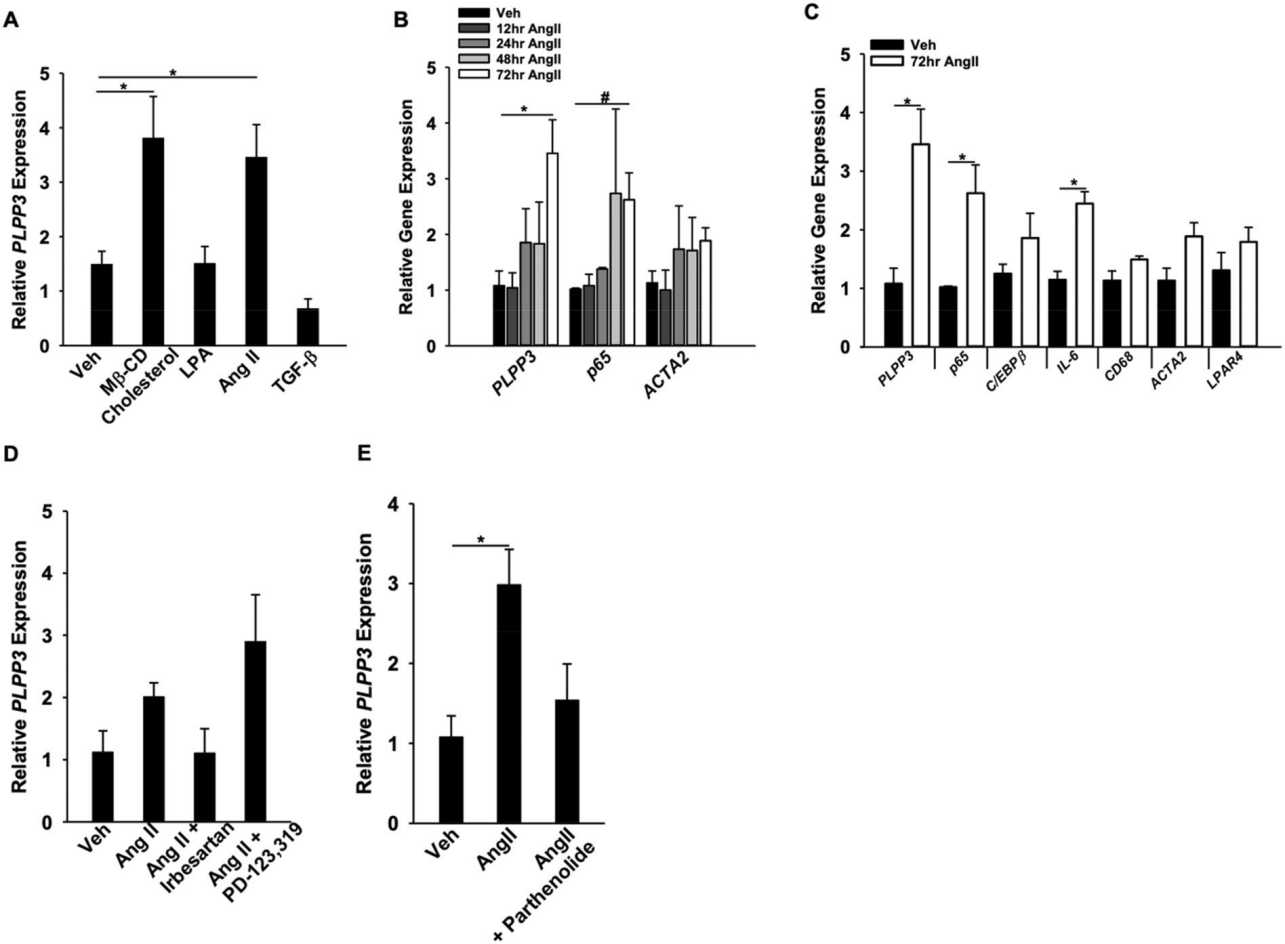
Regulation of *PLPP3* expression by AngII and NF-κB in caHSMC. (**A**) caHSMC were treated with vehicle (n = 3), Mβ-CD Cholesterol (10 μg/ul, n = 3) LPA (1 μM, n = 3)), AngII (1 μM, n = 3), or transforming growth factor beta (TGFβ, 1 μM, n = 3) for 72 h and gene expression analyzed by qRT-PCR using 18 s as internal control. Results are presented as relative gene expression (mean ± SEM), *P < 0.05, One-way ANOVA. (**B**) caHSMCs were treated with vehicle (n = 3) or AngII (1 μM,) for 12 (n = 3), 24 (n = 3), 48 (n = 3) or 72 h (n = 6). Results are presented as relative gene expression (mean ± SEM). *P < 0.05, One-way ANOVA, ^#^*P* < 0.05 Kruskal–Wallis One-way ANOVA on Ranks. (**C**) caHSMCs were treated with vehicle (n = 4) or AngII (1 μM, n = 6) for 72 h. Results are presented as relative gene expression (mean ± SEM), *P < 0.05, unpaired two-tailed student t-test. (**D**) caHSMCs were treated with vehicle, AngII (1 μM), AngII + Irbesartan (1 μM), or AngII + PD-123,319 (1 μM) for 72 h, n = 3 for all treatment conditions. Results are presented as relative *PLPP3* gene expression. (**E**) caHSMCs were treated with vehicle (n = 4), angiotensin II (1 μM, n = 8) or angiotensin II + parthenolide (1 μM, n = 3) for 72 h. Results are presented as relative gene expression (mean ± SEM) *P < 0.05, One-way ANOVA.

Our previous work has demonstrated that LPP3 expression is dynamically upregulated in SMCs under conditions of vascular inflammation and contributes to vascular pathology^4,17,25^. We recently identified RelA and RelB responsive elements in the *PLPP3* gene that regulate expression through canonical activation of NF-kB signaling^19^. We therefore sought to identify upstream regulators of LPP3 expression and observed that AngII, but not LPA or TGF-β, increased LPP3 expression in caHSMCs (Fig. 1A). As previously reported, Mβ-CD cholesterol loading of SMCs also increased LPP3 expression^22^. The AngII effect was time-dependent with highest levels of *Plpp3* mRNA detected at 72 h (Fig. 1B). AngII is a known activator of NF-κB signaling^26^ and *p65* and *IL6* expression, downstream markers of NF-κB signaling, increased at 72 h (Fig. 1B,C). AngII receptor I inhibition blocked the AngII-mediated increase in *PLPP3* gene expression (Fig. 1D), implicating Ang II receptor-mediated signaling. We previously demonstrated that C/EBPβ binding to an enhancer within *PLPP3* upregulates gene expression in response to oxLDL or cholesterol loading^14,22^; however, following AngII treatment of SMC, *C/EBPβ* levels were not significantly altered (Fig. 1C). Instead, the NF-κB signaling inhibitor parthenolide attenuated AngII-induced increases in *PLPP3* gene expression (Fig. 1E), suggesting a role for NF-κB signaling downstream of AngII.

### Loss of *Plpp3* in smooth muscle cells protects against aortic aneurysm and transmural rupture

**Figure 2:**
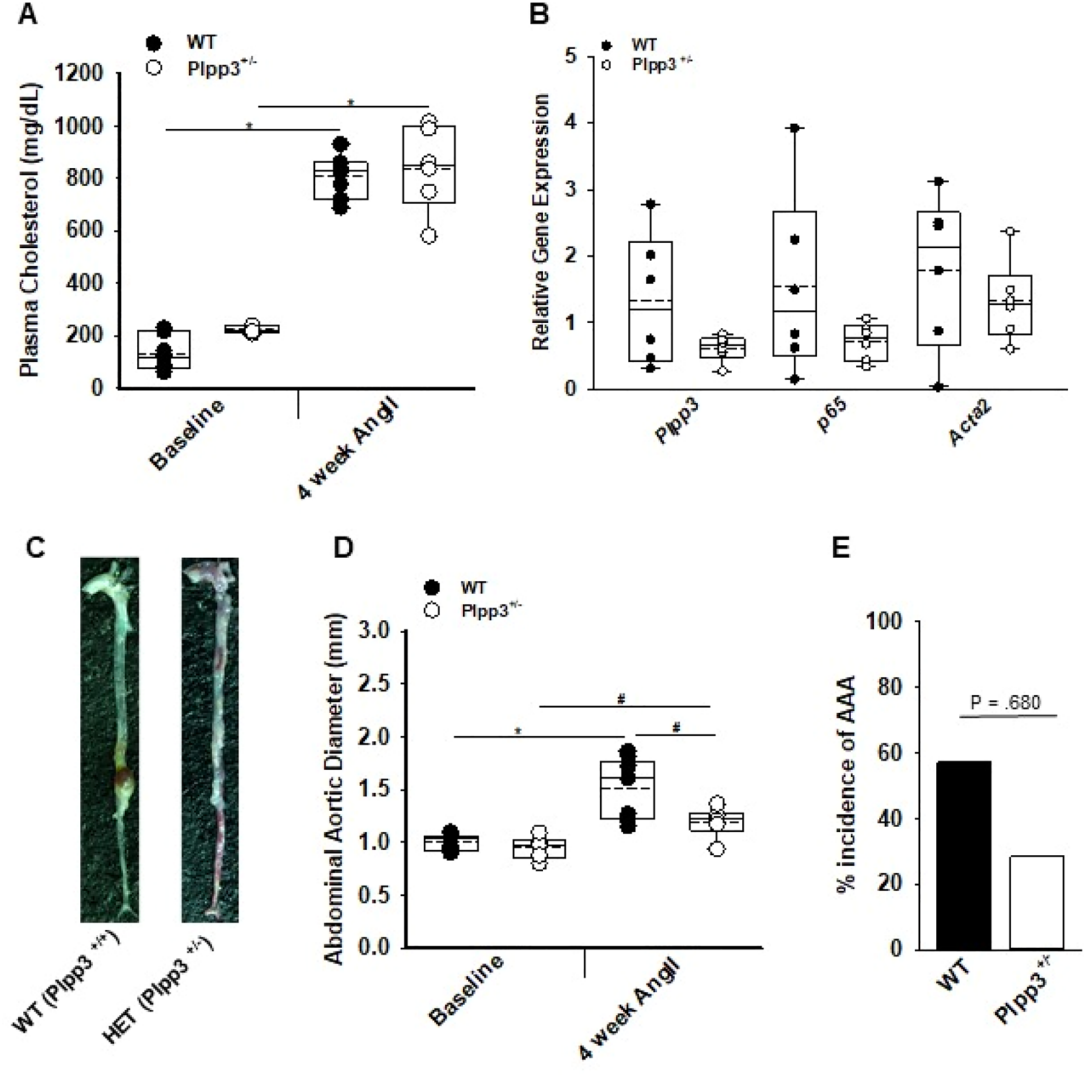
Reduced *Plpp3* expression protects against abdominal aortic aneurysm development. (**A**) Plasma cholesterol at baseline and post-4 week AngII were measured in WT (n = 7) and *Plpp3*^+*/−*^ (n = 6) mice, *P < 0.001, Two-way ANOVA, Bonferroni t-test, all pairwise multiple comparison. (**B**) Abdominal aorta gene expression from WT (n = 6) and *Plpp3*^+*/−*^ (n = 6) following AngII treatment. (**C**) Representative images of aortas from WT and *Plpp3*^+*/−*^. (**D**) Abdominal aortic diameter measurements of WT (n = 9) and *Plpp3*^+*/−*^ (n = 6) were measured using ultrasound at baseline and 4 week AngII,*P < 0.001, ^#^*P* < 0.05, Two-way ANOVA, Holm-Sidak, all pairwise multiple comparison. (**E**) Percent incidence of AAA in WT (n = 9) and *Plpp3*^+*/−*^ (n = 6) mice treated with AngII for 4 weeks *P* = 0.068 by Fischer’s exact test.

In hyperlipidemic mice, AngII (1000 ng/kg/min) stimulates aortic aneurysm formation^27^. Therefore, we sought to determine whether AngII-upregulation of LPP3 influenced pathologic progression by using mice heterozygous for *Plpp3* expression (*Plpp3*^+*/−*^). Hyperlipidemia was achieved by injecting littermate control WT or *Plpp3*^+*/−*^ mice with PCSK9D377Y.AAV to lower LDL receptor expression and feeding the animals Western diet. At 28 days after AngII infusion, plasma cholesterol levels were similar in *Plpp3*^+*/−*^ and WT littermate control animals (Fig. 2A). Abdominal aorta expression of *Plpp3* was lower in *Plpp3*^+*/−*^ as compared to WT mice (Fig. 2B). Interestingly, the overall abdominal aortic diameter was smaller and transmural rupture was decreased in hyperlipidemic *Plpp3*^+*/−*^ mice after AngII infusion (Fig. 2C,D; *P* < 0.05), although there was no difference in the overall incidence of aneurysm formation, as defined by a greater than 50% increase of abdominal aortic diameter from baseline to post AngII (Fig. 2E). No difference was observed in aortic arch diameter between WT and *Plpp3*^+*/−*^ mice (1.923 versus 2.01 mm; *P* = 0.633; student t-test). The small abdominal aortic diameter suggests that LPP3 may normally promote aneurysm formation in the AngII model and that a reduction in LPP3 expression may be sufficient to provide protection.

**Figure 3:**
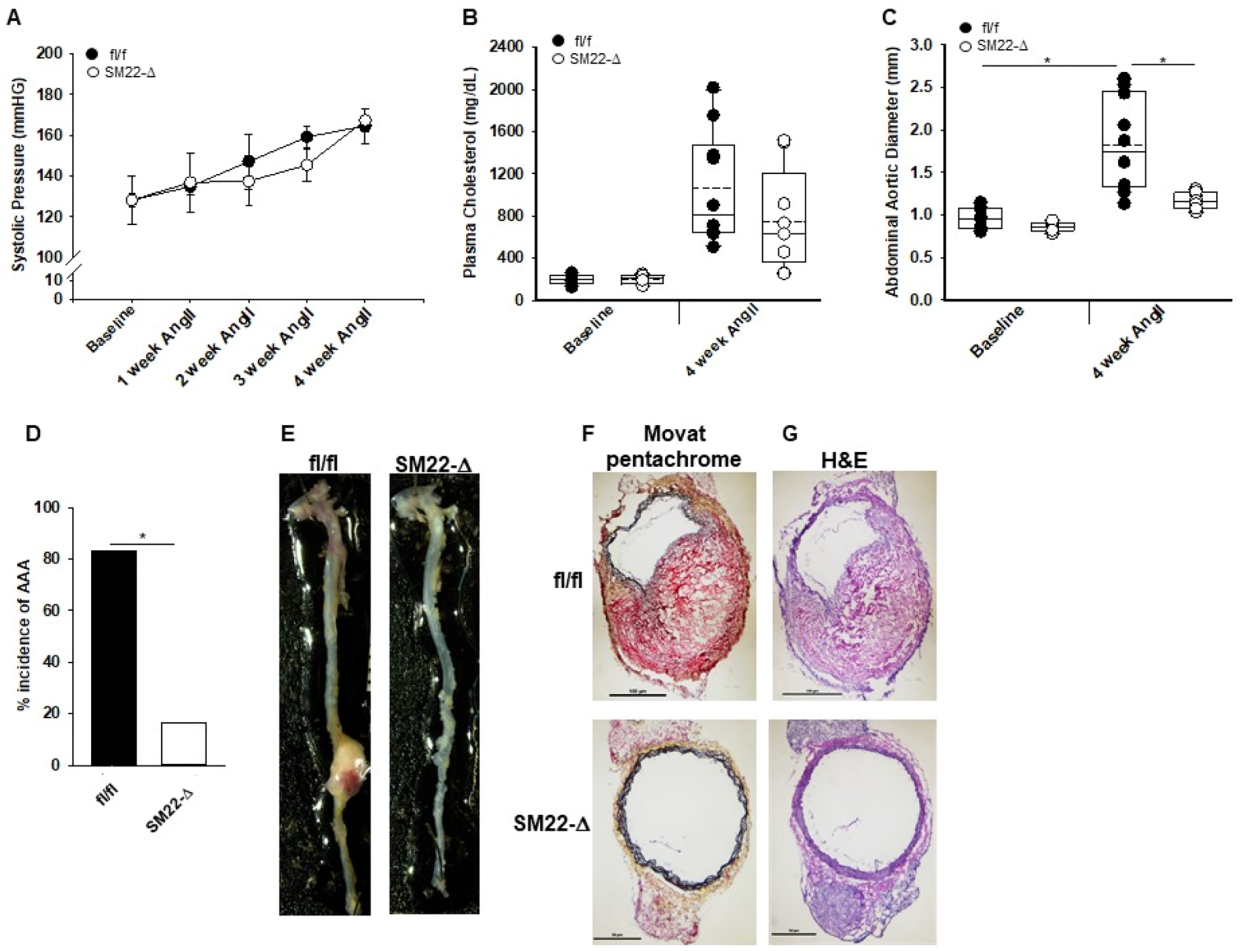
Loss of smooth muscle cell *Plpp3* reduces abdominal aortic aneurysm development. (**A**) Systolic blood pressure measurements from fl/fl (n = 4) and Sm22-Δ (n = 3) mice treated with AngII for 4 weeks. (**B**) Plasma cholesterol at baseline and post-4 week AngII were measured in fl/fl (n = 10) and SM22-Δ (n = 7) mice. (**C**) Abdominal aortic diameter measurements of fl/fl (n = 10) and SM22-Δ (n = 8) were measured using ultrasound at baseline and 4 week AngII. *P < 0.001, Two-way ANOVA, Holm-Sidak, all pairwise multiple comparison. (**D**) Percent incidence of AAA in fl/fl (n = 10) and SM22-Δ (n = 7) mice treated with AngII for 4 weeks. (**E**) Representative images of aortas from fl/fl and SM22-Δ. (**F**) Representative movat pentachrome stained images of abdominal aorta from fl/fl and SM22-Δ mice following 4 week AngII treatment. (**G**) Representative H&E stained images of abdominal aorta from fl/fl and SM22-Δ mice following 4 week AngII treatment.

We next investigated whether SMC expression of LPP3 was required for aneurysm formation in response to AngII using previously characterized mice lacking SMC *Plpp3* expression on the *Ldlr*^*−/−*^ background (SM22-Δ). At 28 days after AngII infusion, systolic blood pressure (Fig. 3A) and plasma cholesterol (Fig. 3B) were similar in SM22-Δ and littermate control fl/fl animals on the *Ldlr*^*−/−*^ background (fl/fl). No difference was observed in plasma triglycerides between fl/fl vs SM22-Δ mice (527 ± 290 vs 299 ± 246; *P* = 0.177). Similar to *Plpp3*^+*/−*^ mice, SM22-Δ mice had a significant reduction in overall abdominal aortic diameter in comparison to fl/fl mice (1.82 ± 0.53 vs 1.28 ± 0.34 mm; *P* < 0.001; Fig. 3C). SM22-Δ mice also had a lower overall incidence of aneurysm formation as defined by a greater than 50% increase of abdominal aortic diameter from baseline to post AngII and decreased transmural rupture. (84% in fl/fl vs 20% in SM22-Δ; Fig. 3D,E). One-month mortality was similar in the groups at 14.3% in fl/fl and 10.5% in SM22-Δ male mice. Female SM22-Δ mice also displayed a smaller mean abdominal aortic diameter following AngII than their fl/fl littermates (Supplemental Figure I). Histologic examination of abdominal aorta with Movat pentachrome staining (Fig. 3F) and H&E (Fig. 3G) staining demonstrated aortic wall rupture, elastic breaks, and thrombus formation in the fl/fl mice, whereas the overall aortic structure of the medial layer was relatively preserved in SM22-Δ mice. A reduction in LPP3 expression was observed in the media of SM22-Δ animals (Supplemental Figure II). Overall, these results indicate that loss of LPP3 within SMCs protects against aortic aneurysm formation and rupture. No significant difference in vessel contraction was observed in fl/fl and SM22-Δ mice (Supplemental Figure III and IV).

### LPP3 regulates SMC phenotypic switching and vascular inflammation

**Figure 4:**
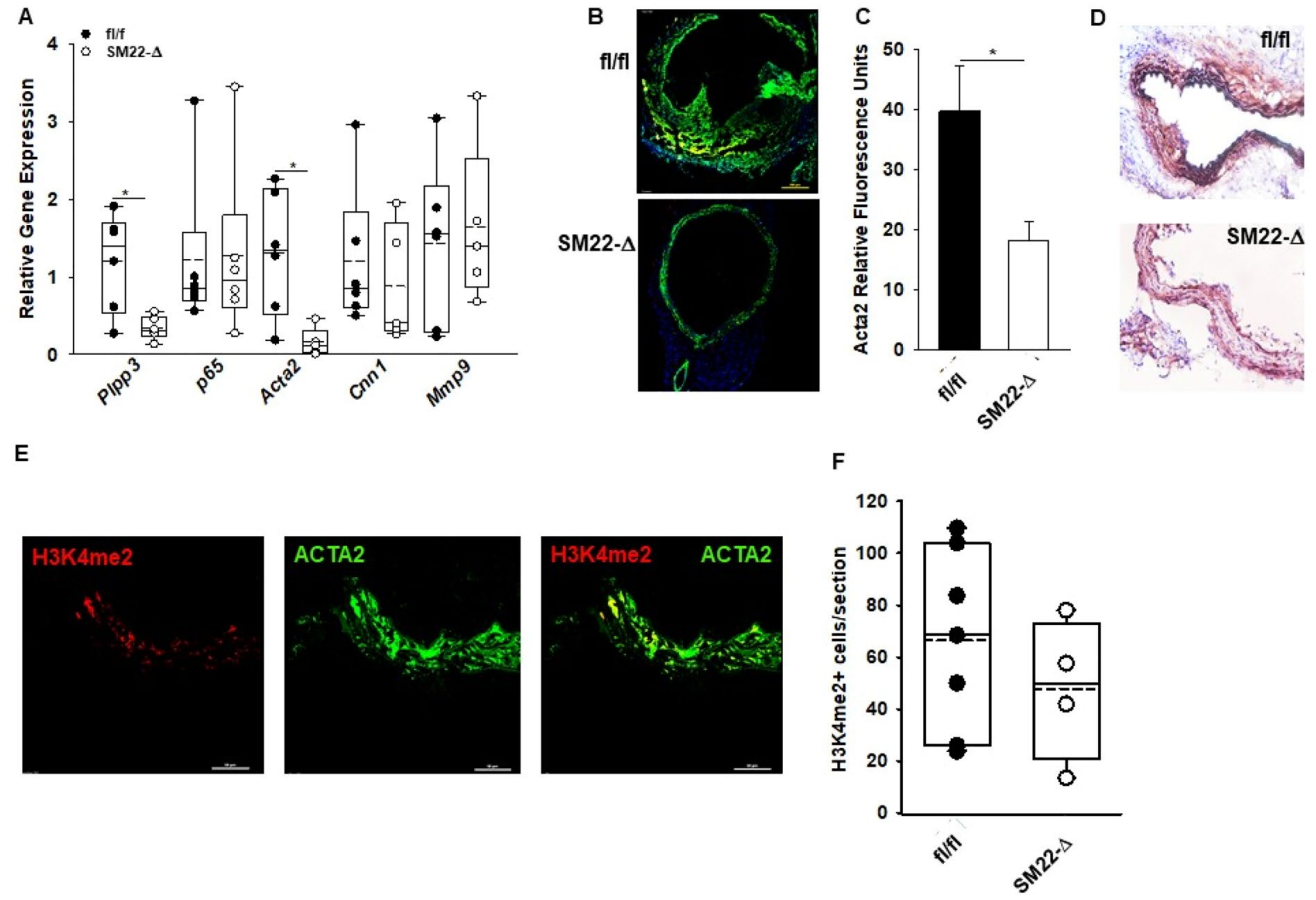
Smooth muscle cell phenotypic switching in SM22-Δ mice. (**A**) Abdominal aorta gene expression from fl/fl (n = 5) and Sm22-Δ (n = 5) mice following 4 week AngII treatment. Data is represented as relative gene expression. Relative gene expression was calculated using an average CT value from fl/fl mice treated with AngII for 4 weeks. fl/fl mice treated with AngII for 4 weeks were used as the control and compared to SM22-Δ mice treated with AngII for 4 weeks. *P < 0.05, unpaired two- tailed student t-test. (**B**) Immunofluorescence of Acta2 (green) and DAPI (blue) in abdominal aorta from fl/fl and SM22-Δ mice following 28 day AngII treatment. (**C**) Relative Acta2 fluorescence in abdominal aorta from fl/fl (n = 7) and SM22-Δ (n = 4) mice treated with AngII. Acta2 staining was quantified with Image J and presented as mean ± SEM. *P < 0.05, Welch’s t-test. (**D**) Representative calponin stained images of abdominal aorta from fl/fl and SM22-Δ mice following 4 weeks AngII treatment. (**E**) Immunofluorescence of H3K4me2 (red) on *Myh11* promoter and Acta2 (green) in abdominal aorta from fl/fl mice following 28 day AngII treatment. Representative image is shown. (**F**) Number of H3K4me2+ cells per section from fl/fl (n = 7) and SM22-Δ (n = 4) mice following AngII treatment.

AngII treatment results in changes in the extracellular matrix that likely result in aortic aneurysm formation. These changes are associated with phenotypic modulation of SMCs resulting in their migration, clonal proliferation, and aneurysm formation^28^. We therefore examined the effect of LPP3 on expression of genes associated with SMC phenotypic modulation, extracellular matrix, and inflammation. At 28 days after AngII infusion, a significant reduction in *Acta2* expression with no change in *p65, Cnn1,* or *Mmp9* occurred in abdominal aortas of SM22-Δ mice (Fig. 4A). In keeping with the gene expression data, ACTA2 staining (Fig. 4B) was also significantly reduced in SM22-Δ compared to fl/fl abdominal aorta at four weeks after AngII infusion (Fig. 4C), along with reduction in calponin expression another marker of differentiated SMC (Fig. 4D), suggesting a difference in phenotypic switching in the SM22-Δ mice. In addition to phenotypic switching, SMCs undergo apoptosis during aneurysm development. To determine if the reduction in *Acta2* and calponin expression reflected a lack of committed SMCs, ISH-PLA was performed on aortic sections to measure dimethylation of lysine 4 on histone 3 (H3K4me2) at the smooth muscle myosin heavy chain 11 locus, an epigenetic mark exclusive to SMC^24^ that is maintained in SMC that undergo phenotypic switching. ISH-PLA for H3K4me2 in fl/fl and SM22-Δ (Fig. 4E) abdominal aorta sections revealed a similar number of positive cells per section (Fig. 4F) suggesting the number of SMCs within the abdominal aorta is similar between fl/fl and SM22-Δ mice. Together this data suggests that the loss of LPP3 in SMCs reduces expression of committed markers such as smooth muscle α-actin and calponin following AngII treatment.

**Figure 5:**
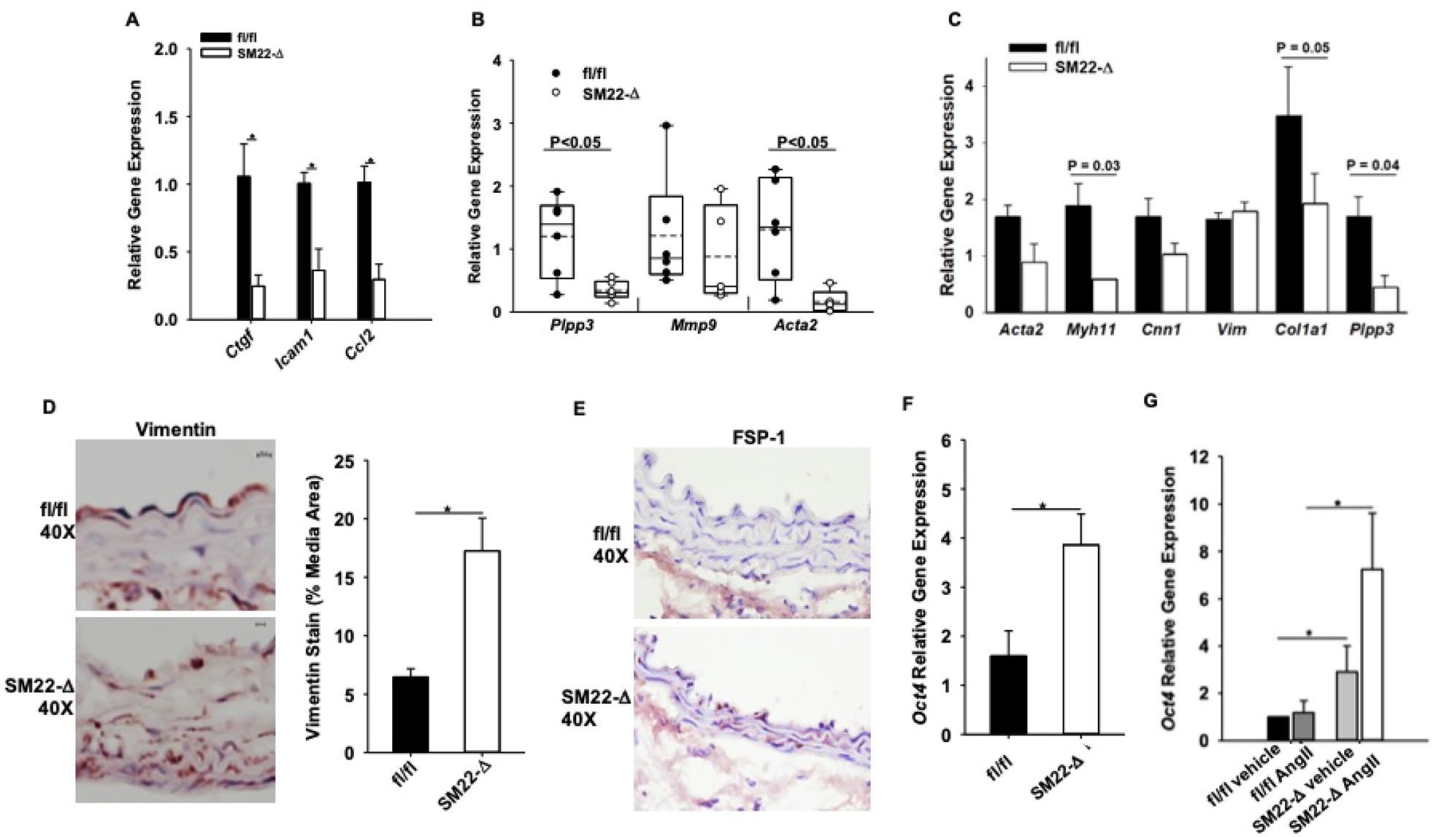
Reduced inflammation in SM22-Δ abdominal aorta. (**A**) Gene microarray were performed in abdominal aorta following 1 week AngII infusion in fl/fl (n = 3) and SM22-Δ (n = 3) mice, *P < 0.05, unpaired two-tailed student t-test. Relative gene expression was calculated using an average CT value from fl/fl mice treated with AngII for 1 week. fl/fl mice treated with AngII for 1 week were used as the control and compared to SM22-Δ mice treated with AngII for 1 week. (**B**) Abdominal aorta gene expression from fl/fl (n = 4) and SM22-Δ (n = 3) mice following AngII treatment. Data is represented as relative gene expression, *P < 0.05, unpaired two-tailed student t-test. Relative gene expression was calculated using an average CT value from fl/fl mice treated with AngII for 1 week. fl/fl mice treated with AngII for 1 week were used as the control and compared to SM22-Δ mice treated with AngII for 1 week. (**C**) Gene expression of isolated aortic SMC from fl/fl (n = 3) and SM22-Δ (n = 3) following 24 h AngII treatment. Relative gene expression was calculated using an average CT value from fl/fl mice treated with AngII for 1 week. fl/fl mice treated with AngII for 1 week were used as the control and compared to SM22-Δ mice treated with AngII for 1 week. (**D**) Representative vimentin stained abdominal aorta from fl/fl andSM22-Δ mice following 1 week AngII infusion and percent area of vimentin stain in abdominal aortas from fl/fl (n = 4) and SM22-Δ (n = 3). Vimentin stain was quantified with Image J and presented as mean ± SEM. *P < 0.05, unpaired two-tailed student t-test. (**E**) Representative FSP-1 stained abdominal aorta from fl/fl and SM-Δ mice following 1 week AngII infusion. (**F**) Abdominal aorta Oct4 gene expression from fl/fl (n = 4) and SM22-Δ mice (n = 4) *P < 0.05, unpaired two-tailed student t-test. Relative gene expression was calculated using an average CT value from fl/fl untreated SMCs. fl/fl untreated SMCs were used as the control and compared to untreated SM22-Δ SMCs. (**G**) Oct4 gene expression of isolated aortic SMC from fl/fl (n = 3) and SM22-Δ (n = 3) following 24 h AngII treatment, *P < 0.05, Two way ANOVA, Holm-Sidak, all pairwise multiple comparison. Relative gene expression was calculated using an average CT value from fl/fl 24 h AngII treated SMCs. fl/fl 24 h AngII treated SMCs were used as the control and compared to 24 h AngII SM22-Δ SMCs.

To determine if LPP3 was regulating early changes in the aortic wall prior to aneurysm formation, mice were treated with AngII for one week, a time point in which abdominal diameters were the same in fl/fl and SM22-Δ mice as measured by ultrasound (Supplemental Figure V). At one week after AngII infusion, plasma cholesterol (738 ± 176 vs 749 ± 257 mg/dL; *P* = 0.946) and triglycerides (323 ± 115 vs 356 ± 207 mg/dL; *P* = 0.779) were not significantly different between fl/fl vs SM22-Δ animals. Movat pentachrome and H&E staining revealed an overall similar aortic structure between genotypes with no difference in elastic breaks (Supplemental Figure V), adventitial thickening (0.33 ± 0.19 vs 0.17 ± 0.02 µm^2^; *P* = 0.276), medial thickening (0.346 ± 0.1.16 vs 0.188 ± 0.101 µm; *P* = 0.101), TUNEL staining (Supplemental Figure V), CD68 staining (Supplemental Figure VI), collagen hydrolyzing peptide (Supplemental Figure VII), or picrosirius red staining (Supplemental Figure VIII) in fl/fl and SM22-Δ mice at this early time point. Despite these histologic similarities, gene array expression data demonstrated lower levels of *Ctgf*, *Icam1*, and *Ccl2* in SM22-Δ tissue (Fig. 5A), consistent with alterations in inflammation and extracellular matrix pathways following AngII treatment. Additionally, *Acta2* expression was significantly decreased with no change in *Mmp9* in SM22-Δ abdominal aortas one week after AngII treatment (Fig. 5B).

To understand whether these differences reflected alterations in the phenotypic state of SMCs, aortic SMCs were isolated from fl/fl and SM22-Δ mice and treated with AngII in culture. As expected, *Plpp3* expression was significantly lower in SM22-Δ than fl/fl cells (Fig. 5C) following AngII treatment. SMCs from SM22-Δ mice had significantly reduced levels of Myh11 and Col1a1 with a trend of decreasing Acta2 gene expression (Fig. 5C). While vimentin gene expression was not increased in isolated SMCs from SM22-Δ mice following AngII treatment, vimentin staining (Fig. 5D) was increased within the medial layer of aortas from SM22-Δ mice following 1 week AngII infusion, suggesting a fibroblast-like phenotype. In keeping with these observations, FSP-1 (Fig. 5E) staining was higher within the medial layer of aortas from SM22-Δ mice. Recently, the pluripotency factor Oct4 has been demonstrated to regulate SMC function and phenotypic switching^29^. We observed significant upregulation of Oct4 mRNA in abdominal aorta of control SM22-Δ mice (Fig. 5F) and isolated aortic SMC from SM22-Δ mice (Fig. 5G). These findings suggest that in the absence of LPP3, SMC have lower expression of SMC markers and higher vimentin, FSP-1 and Oct4, suggesting changes in regulation of SMC differentiation state.

**Figure 6:**
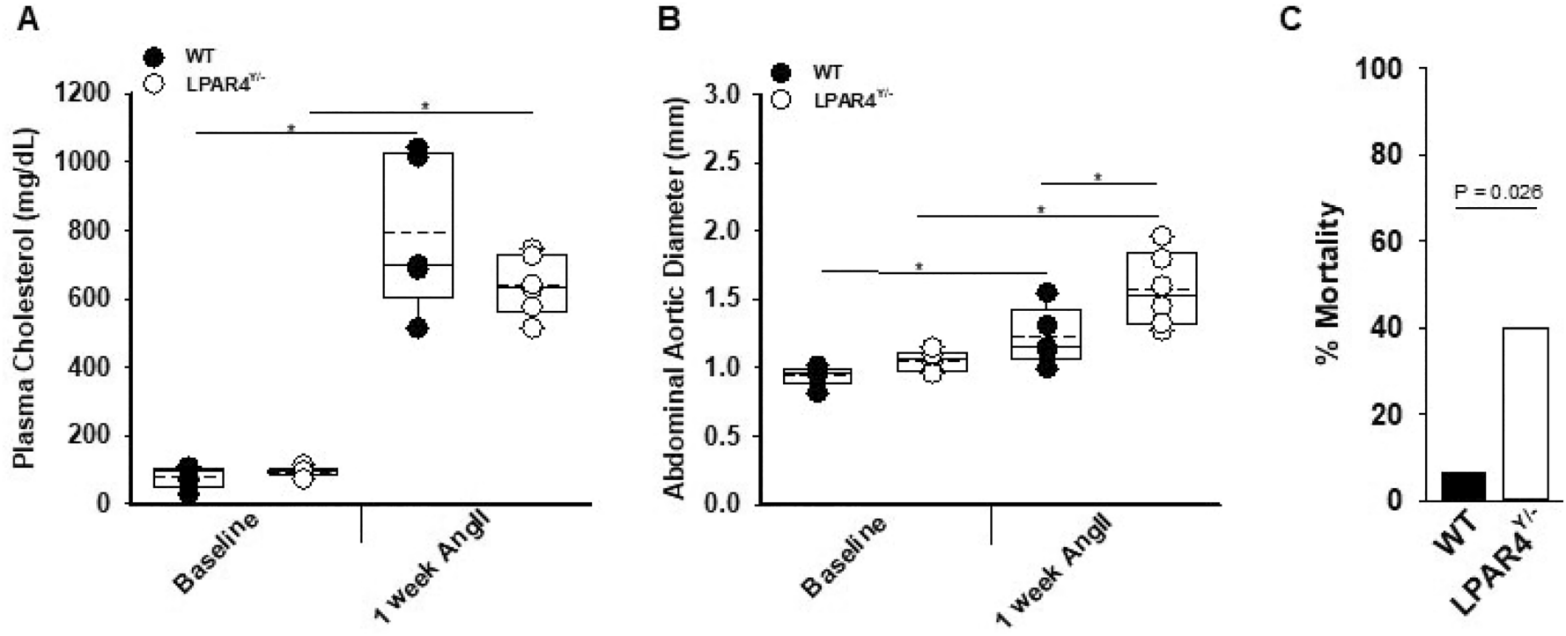
Loss of lysophosphatidic acid receptor 4 increases abdominal aortic aneurysm formation. (**A**) Plasma cholesterol at baseline and post-1 week AngII were measured in WT (n = 5) and *LPAR4*^*Y/−*^ (n = 6) mice, *P < 0.001 Two way ANOVA, Bonferroni t-test, all pairwise multiple comparison. (**B**) Abdominal aortic diameter measurements of WT (n = 5) and *LPAR4*^*Y/−*^ (n = 6) were measured using ultrasound at baseline and 1 week AngII. *P < 0.05, Two way ANOVA, Holm-Sidak, all pairwise multiple comparison. (**C**) Percent mortality of WT (n = 16) and *LPAR4*^*Y/−*^ (n = 20) mice within one week of treatment with AngII; *P* = 0.026 by Fisher’s exact test.

LPP3 is known to regulate LPA signaling^22,30–32^, which in turn promotes SMC de-differentiation^33^ and fibrosis^34–36^. Indeed, a reduction in LPP3 expression will lower LPA degradation and could potentiate the effect of LPA on de-differentiation and fibrosis, consistent with the phenotype observed in the SM22-Δ mice. If LPP3 is normally limiting the effects of LPA, then we would expect that LPA signaling may provide protection against the development of dissecting aneurysm in response to AngII. In mice, LPAR4 has been implicated in vascular development and inflammation in the context of atherosclerosis^21^, and therefore, we examined the phenotype of mice lacking LPAR4 signaling in the AngII-aneurysm model. For these experiments, LPAR4 deficient mice (*LPAR4*^*Y/−*^) or WT controls were treated with PCSK9D377Y.AAV and fed Western diet to elicit hyperlipidemia. Plasma cholesterol levels were similar at 1 week after AngII infusion (Fig. 6A). Abdominal aortic diameter was significantly greater in *LPAR4*^*Y/−*^ mice compared to WT (Fig. 6B). Additionally, the *LPAR4*^*Y/−*^ mice were more prone to death due to rupture with an odds ratio of > 9 (Fig. 6C; *P* = 0.026). In the surviving mice, plasma cholesterol, abdominal aortic dimensions, *Plpp3* and *Acta2* gene expression were similar between WT and *LPAR4*^*Y/−*^ mice at four weeks (Supplemental Figure IX). The early increase in abdominal diameters and increased rupture rate in mice lacking LPAR4 is consistent with the hypothesis that heightened LPA signaling in context of LPP3 deficiency contributes to protection from aneurysm formation.

## Discussion

Abdominal aortic aneurysm (AAA) is a potentially fatal condition due to aortic rupture, with a 4–7% prevalence in men over 55 years old^37^. Currently, monitoring aneurysm growth followed by surgical intervention represents the only treatment option for AAA^38^. The pathophysiology of AAA is complex and not completely understood but includes macrophage accumulation, alterations in inflammatory mediators, changes in extracellular matrix integrity, and smooth muscle cell phenotypic switching^38^.

In this study, we provide the first evidence that lysophospholipid signaling regulates the development of dissecting aortic aneurysm elicited by AngII in hyperlipidemic mice and suggest novel targets for therapeutic intervention. Specifically, the loss of LPP3 in SMC confers protection against the formation of dissecting abdominal aortic in association with significant reductions in SMC makers, inflammatory markers and a corresponding increase in fibroblast markers. We previously demonstrated that LPP3 regulates SMC differentiation following vascular injury in carotid arteries or in the context of atherosclerosis. Somewhat surprisingly, while the loss of LPP3 in SMC increased the development of atherosclerosis in mice, it protects from AngII-mediated aneurysm formation. The role of LPP3 in regulating the development of dissecting aortic aneurysm may reflect a role in influencing phenotypic switching within SMC and the associated changes in the SMC contractile apparatus.

LPP3 degrades and thereby inactivates the bioactive lipid LPA, which is a potent inducer of SMC de-differentiation^17^ and a pro-fibrotic mediator. We have demonstrated that LPP3 controls LPA levels, LPA signaling^17,39^ and that the absence of LPP3 increases LPA receptor mediated signaling in SMC. Thus, it is possible that the effects of LPP3 on SMC differentiation markers are mediated by regulating local levels of bioactive LPA in the context of aneurysm formation. In the absence of LPP3, excessive LPA signaling could result in exaggerated de-differentiation of SMC and their conversion to a more fibroblast-like phenotype that provides protection against aneurysm formation. In support of this hypothesis, the loss of the LPA specific LPAR4 receptor worsens AngII-induced abdominal aortic expansion and increases overall mortality associated with rupture. These findings indicate that LPA signaling plays a protective role in this model of AAA, which is in contrast to the permissive effect of LPA signaling on experimental atherosclerosis.

It is important to note that *Sm22* expression is not limited to smooth muscle cells^40^ so it is possible other cell types could be responsible for the reduction in AAA seen in the SM22-Δ mice. CD68 staining, a macrophage marker, revealed no difference between fl/fl and SM22-Δ mice indicating any potential loss of LPP3 from myeloid cells due to off targeted effects of the SM22 Cre did not impact macrophage migration into aortic tissue.

In other models, de-differentiation of SMC promotes aneurysm formation^41,42^ and atherosclerosis burden^43^. Indeed, our own results demonstrate that the loss of LPP3 in SMC promotes atherosclerosis at the aortic root and arch. It is possible that the protection from aneurysm despite SMC de-differentiation observed in this study reflects a difference in the mechanism of formation (elastase vs AngII) and/or differences in embryonic lineage of SMCs within the aorta. Aortic root and aortic arch SMCs derive from the secondary heart field and cardiac neural crest, respectively, while abdominal aortic SMCs arise from somites^44^. Differences in embryonic origin of the SMC may also contribute to phenotypic differences in the atherosclerosis and aneurysm models. Finally, while the pathophysiology of atherosclerosis and aneurysm are similar there are notable examples of discordance in their development, such as occurs in diabetics who are predisposed to atherosclerotic vascular disease and protected from AAA; likewise, testosterone^45^ and the interferon gamma system^46^ have discordant effects on development of atherosclerosis and aneurysm.

Our previous work^17^ and supplemental Figure III, IV show no change in baseline vasculature vessel function in SM22-Δ, despite consistent gene expression indicating SMC phenotyping switching at baseline. Recent single cell RNA-Seq work has shown that SMC phenotypic switching lies on a continuum during disease progression^47^ and we hypothesize that the loss of Lpp3 in SMCs promotes phenotypic switching but prevents progression of SMC phenotyping switching that is detrimental in disease states. This may reflect the ischemic pre-conditioning model in cardiac injury in which small amounts of ischemia protect against subsequent ischemic events^48^. Future work examining the progression of SMC phenotyping switching during AAA and single cell RNA-Seq cell clustering will aid in our understanding of how SMC phenotyping switching in SM22-Δ mice may differ to provide protection against AAA.

Of important clinical relevance, if heightened LPA signaling contributes to the protection from dissecting aortic aneurysm, then inhibitors of autotaxin, the enzyme responsible for bioactive LPA generation, could promote the pathology. At present, autotaxin inhibitors are in phase 3 clinical trials for efficacy in treating idiopathic pulmonary fibrosis^49^ and should be monitored cautiously for an increase in risk for aortic aneurysm rupture.

A limitation of our study is the use of AngII to promote the development of aneurysm formation. While this model shares some features with AAA development in humans, it does not faithfully recapitulate pathology that occurs in humans. Indeed, some have proposed that it is a better model of aortic dissection than aneurysm^27^. Additionally, small samples size and wide variation in gene expression profiles for some genes limit the conclusions that can be made; however, consistent changes in *Acta2* in smooth muscle deficient Lpp3 mice highlight a role for smooth muscle Lpp3 in AngII mediated abdominal aortic aneurysm. Further studies will need to address the exact mechanism by which Lpp3 loss in smooth muscle protects against abdominal aortic aneurysm.

In summary, we demonstrate a novel pathway for regulation of LPP3 during vascular inflammation involving a NF-κB dependent pathway. SMC LPP3 regulates phenotypic modulation, and in the absence of LPP3, SMCs assume a more de-differentiated fibroblast-like phenotype that is associated with protection from the development of abdominal aortic aneurysm following AngII infusion. Loss of LPAR4 promotes early aneurysmal dilation and rupture, indicating that LPA signaling normally protects against AngII-induced pathology. These findings provide important information about novel pathways that regulate SMC phenotype in the context of pathologically important processes.

## Supplementary Information

Supplementary Information.
