# Supplementary material for: Lipid phosphate phosphatase 3 in smooth muscle cells regulates angiotensin II-induced abdominal aortic aneurysm formation
Source: Sci Rep. 2022 Apr 5;12:5664. doi: 10.1038/s41598-022-08422-7 (PMC8983654; doi:10.1038/s41598-022-08422-7)
Supplement: Supplementary file 1 — Supplementary Information. [file 41598_2022_8422_MOESM1_ESM.docx]

**Lipid Phosphate Phosphatase 3 in Smooth Muscle Cells Regulates Angiotensin II-Induced Abdominal Aortic Aneurysm Formation**

Patrick M. Van Hoose, Liping Yang, Maria Kraemer, Margo Ubele, Andrew J. Morris, Susan S. Smyth

**Supplemental Figures**


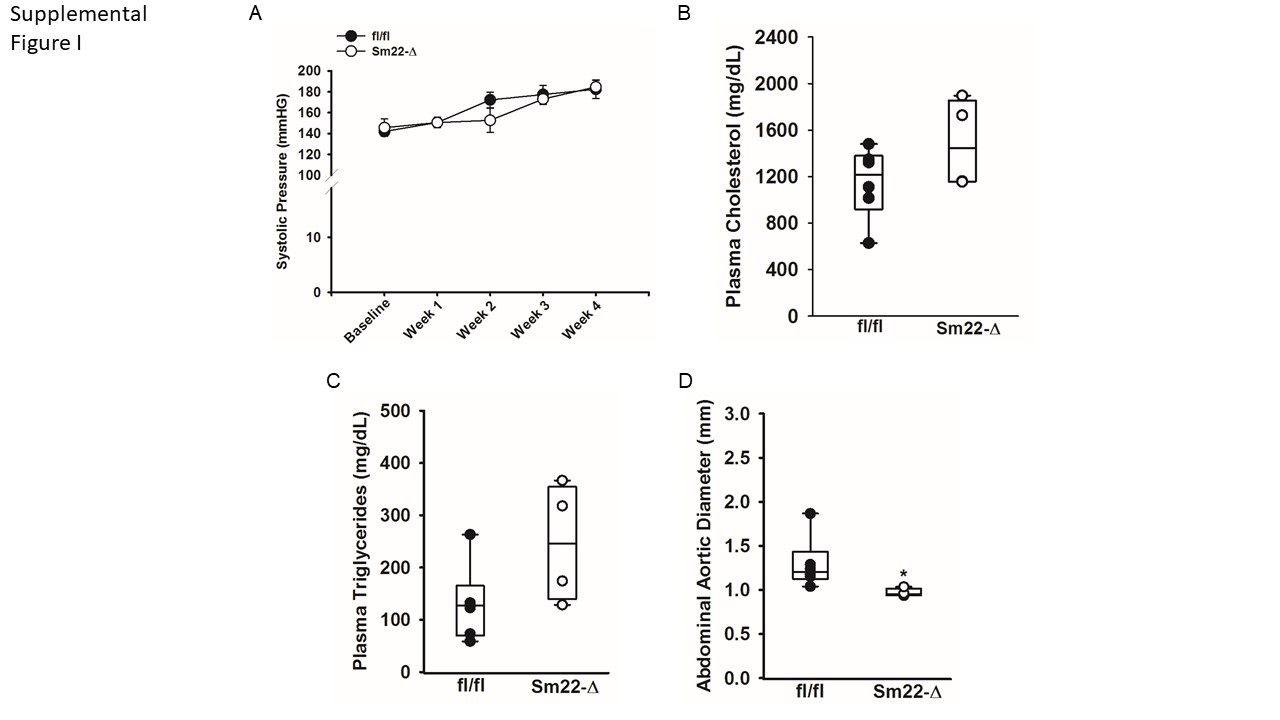


**Supplemental Figure I.** Abdominal aortic aneurysm is reduced in female mice lacking smooth muscle Lpp3. **A**, Systolic blood measurements from fl/fl (n=6) and SM22-Δ (n=4) mice treated with AngII for 4 weeks. **B**, Plasma cholesterol post-4 week AngII were measured in fl/fl (n=6) and SM22-Δ (n=4) mice. **C**, Plasma triglycerides post-4 week AngII were measured in fl/fl (n=6) and SM22-Δ (n=4) mice. **D**, Abdominal aortic diameter measurements of fl/fl (n=6) and SM22-Δ (n=4) were measured post 4 week AngII, *P<0.05, Mann-Whitney Rank Sum Test.


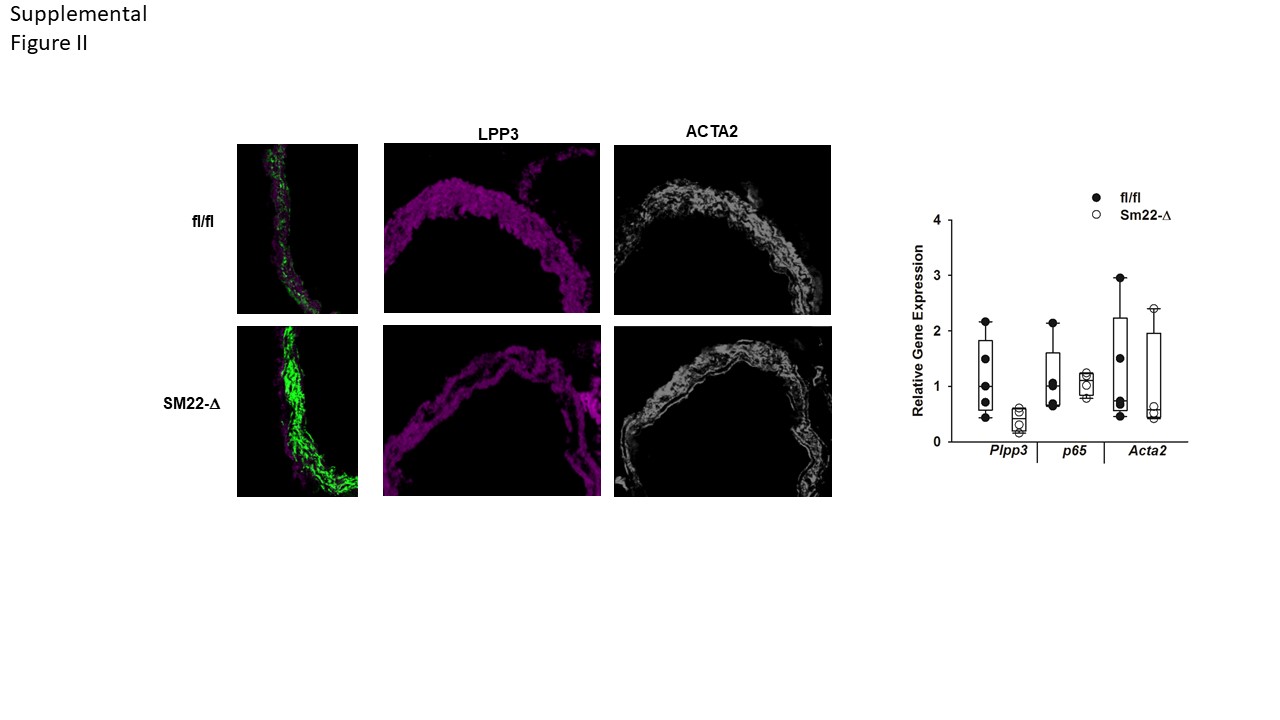


**Supplemental Figure II.** Expression of LPP3 and ACAT2 (left) and gene expression (right, relative gene expression, fl/fl vs SM22-Δ following 1 week AngII treatment) in abdominal aorta from fl/fl and SM22-Δ mice one week after AngII infusion.


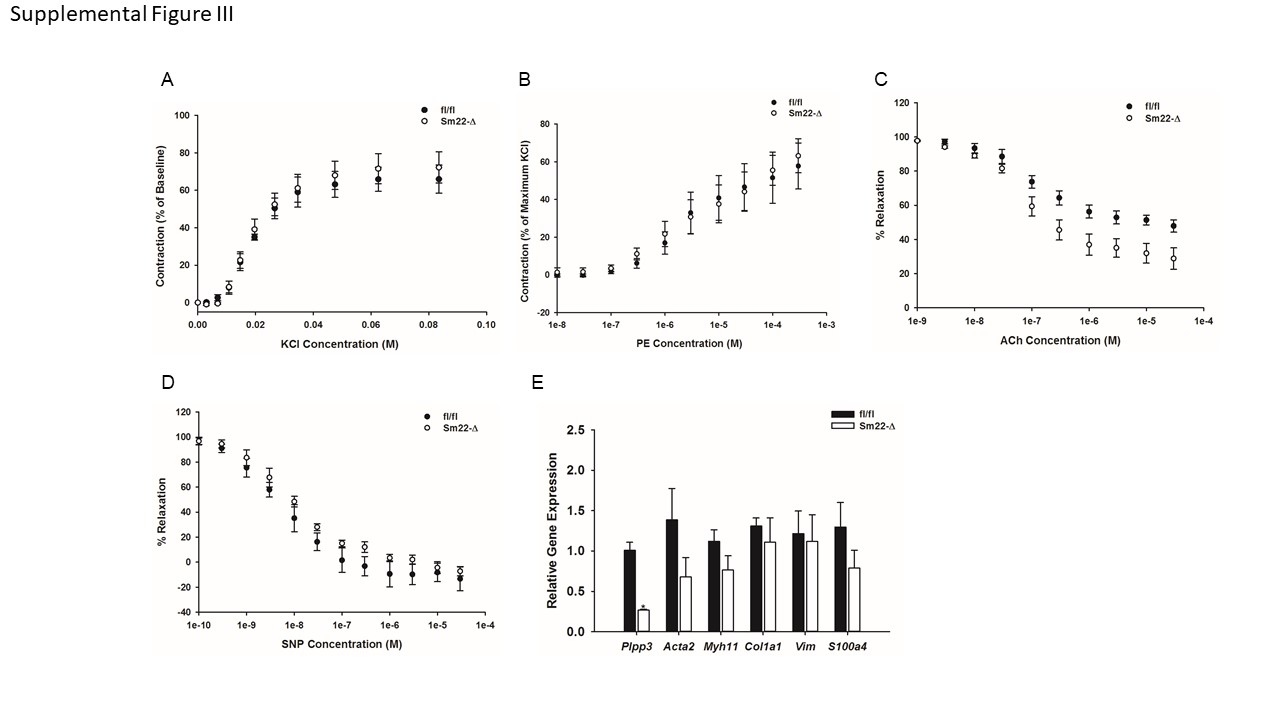


**Supplemental Figure III.** Thoracic aorta vessel reactivity. **A**, Potassium chloride (KCl) concentration-response curve comparing fl/fl and SM22-Δ mice. Data is represented as contraction percent of baseline. **B**, Phenylephrine (PE) concentration-response curve comparing fl/fl and SM22-Δ mice. Data is represented as percent contraction normalized to maximum KCl. **C**, Acetylcholine (ACh) concentration-response curve comparing fl/fl and SM22-Δ mice. Data is represented as percent relaxation. **D**, Sodium nitroprusside (SNP) concentration-response curve comparing fl/fl and SM22-Δ mice. Data is represented as percent relaxation. **E**, Baseline abdominal aorta gene expression from fl/fl and SM22-Δ mice. Data is represented as relative gene expression (untreated fl/fl vs untreated SM22- mice)


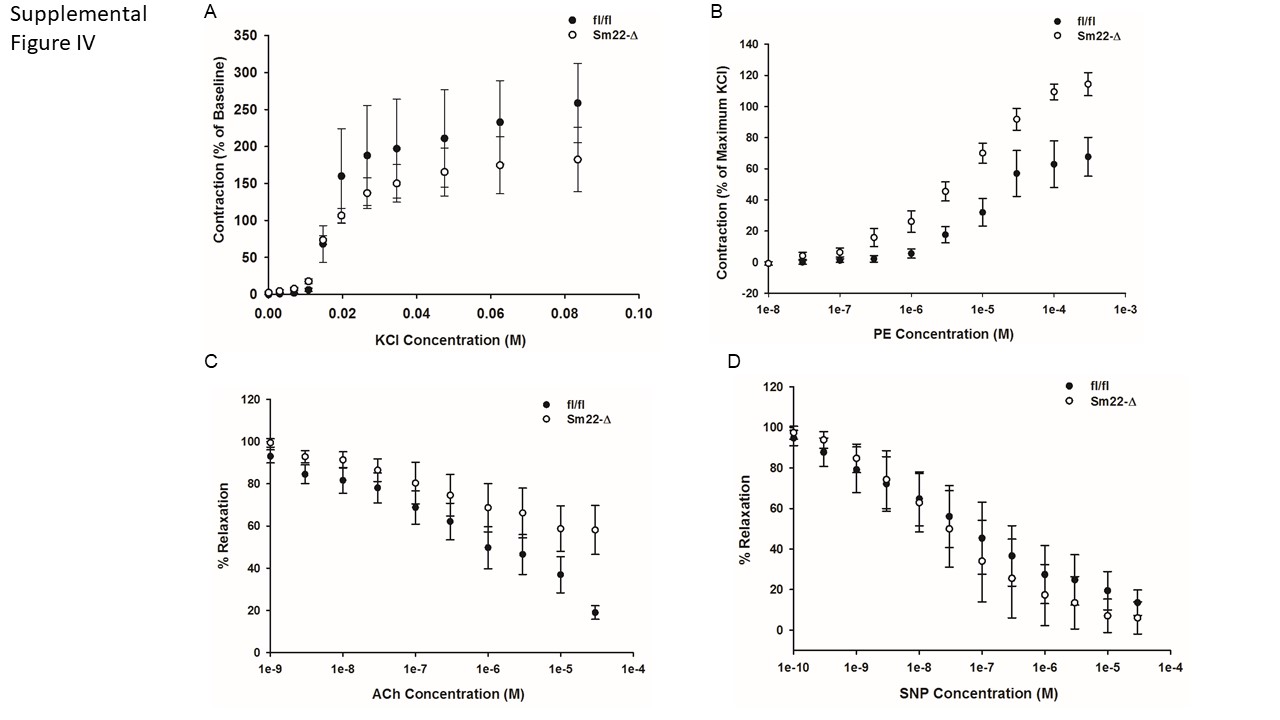


**Supplemental Figure IV**. Mesenteric artery vessel reactivity. **A**, Potassium chloride (KCl) concentration-response curve comparing fl/fl and SM22-Δ mice. Data is represented as contraction percent of baseline. **B**, Phenylephrine (PE) concentration- response curve comparing fl/fl and SM22-Δ mice. Data is represented as percent contraction normalized to maximum KCl. **C**, Acetylcholine (ACh) concentration-response curve comparing fl/fl and SM22-Δ mice. Data is represented as percent relaxation. **D**, Sodium nitroprusside (SNP) concentration-response curve comparing fl/fl and SM22-Δ mice. Data is represented as percent relaxation.


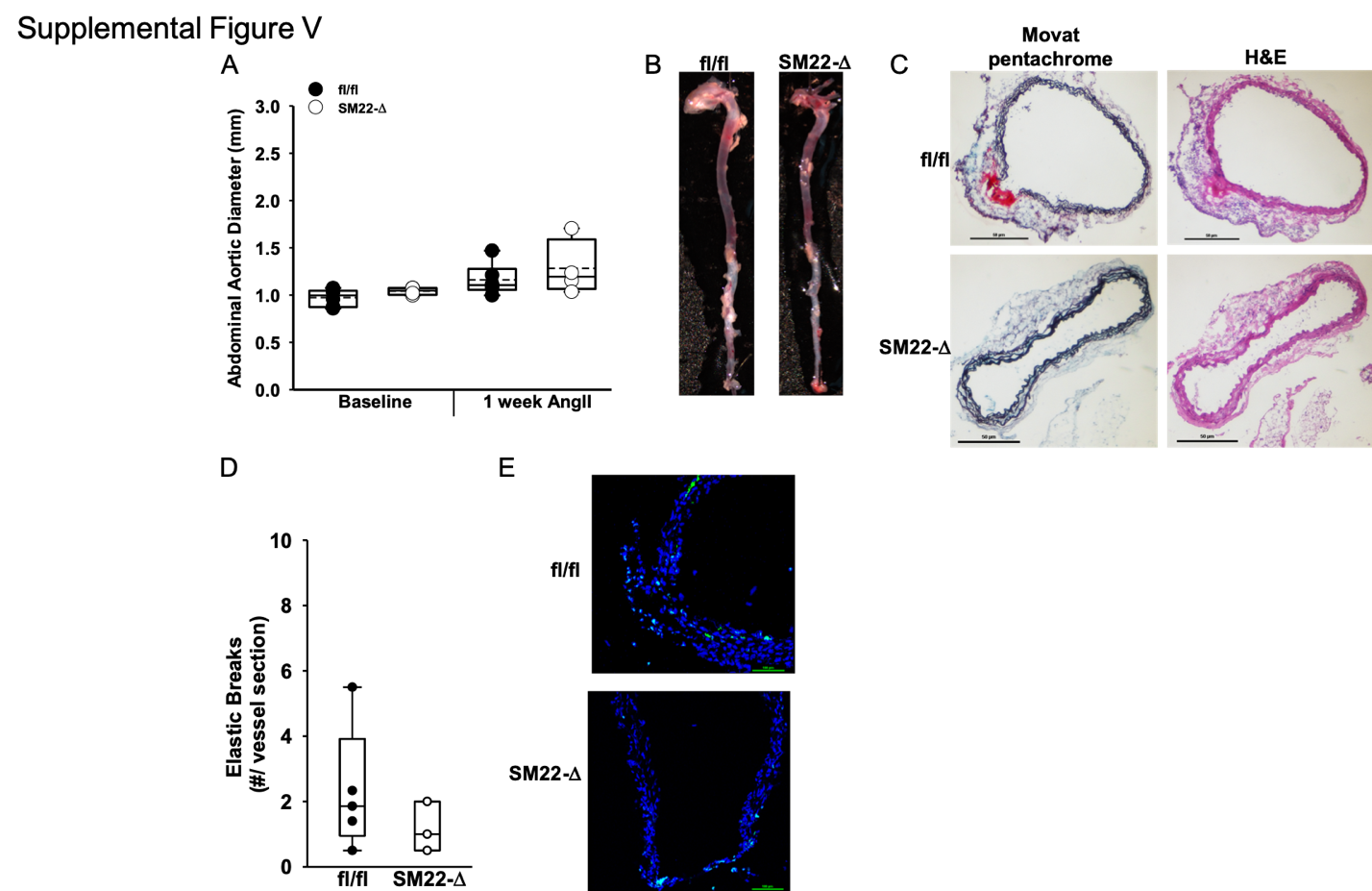


**Supplemental Figure V**. Abdominal aortic structure at one week after AngII in mice lacking smooth muscle *Plpp3*. **A**, Ultrasound measurements from fl/fl and SM22-Δ mice at baseline and post-1 week AngII. **B,** Representative aortas from fl/fl and SM22-Δ mice following 1 week AngII. **C**, Representative movat pentachrome and H&E stained images of abdominal aorta from fl/fl and SM22-Δ mice following 1 week AngII infusion. **D**, Elastic breaks quantified in fl/fl and SM22-Δ mice. Results are represented as number of breaks per vessel section. **E**, TUNEL staining (dark blue DAPI, light blue TUNEL+ cells) in fl/fl and SM22-Δ mice abdominal aorta following 1 week AngII treatment


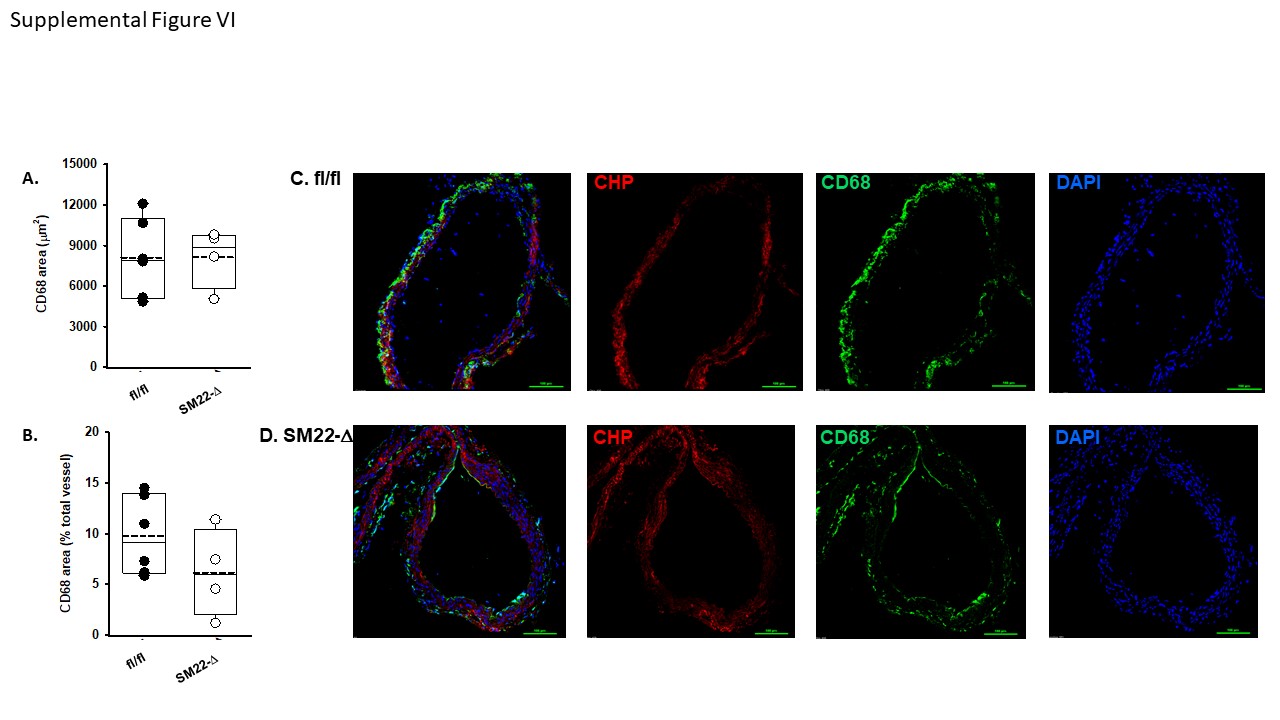


**Supplemental Figure VI**. Macrophage recruitment into abdominal aorta. **A**, CD68 area in fl/fl (n=6) and SM22-Δ (n=4) abdominal aorta following 1 week AngII treatment. **B**, CD68 area (percent of vessel area) in fl/fl and SM22-Δ abdominal aorta following 1 week AngII treatment. **C**, Representative CD68 and DAPI stained abdominal aorta from fl/fl mice following 1 week AngII. **D**, Representative CD68 and DAPI stained abdominal aorta from SM22-Δ mice following 1 week AngII.


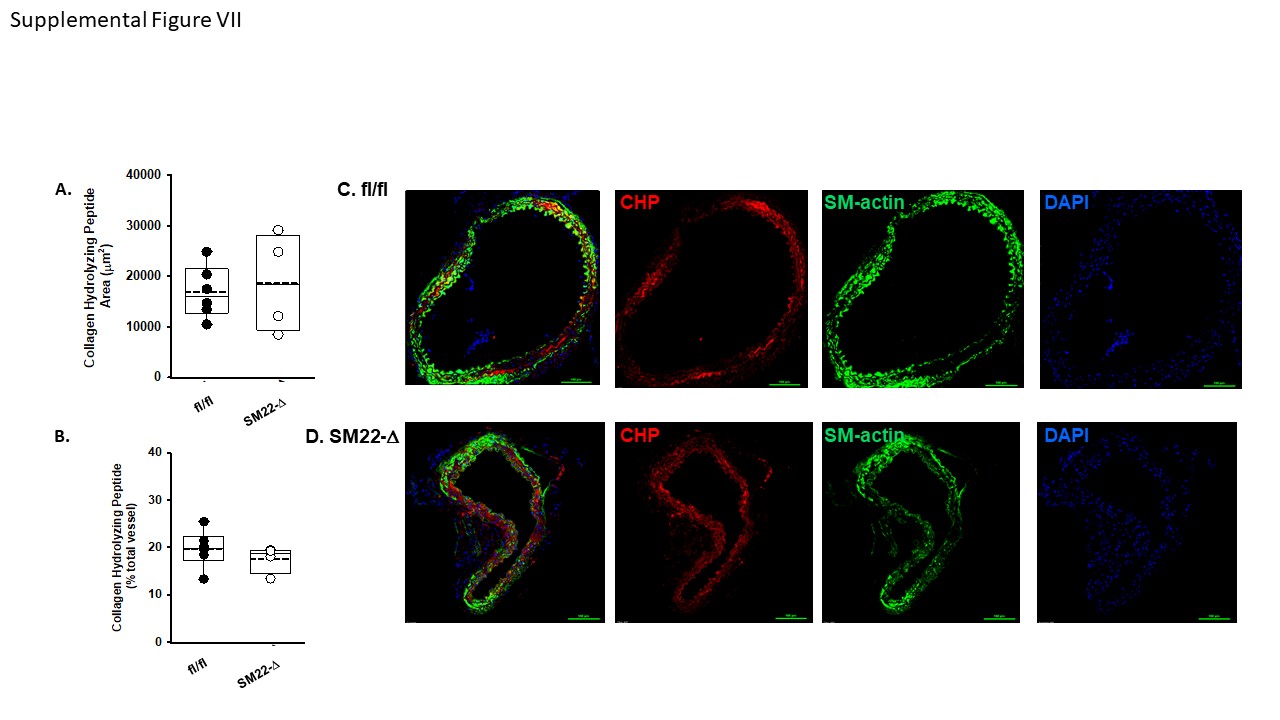


**Supplemental Figure VII**. Collagen degradation in abdominal aortic vessels. **A**, Collagen hydrolyzing peptide (CPH) area in fl/fl (n=4) and SM22-Δ (n=4) abdominal aorta following 1 week AngII treatment. **B**, Collagen hydrolyzing peptide (percent of total vessel area) in fl/fl (n=4) and SM22-Δ (n=4) abdominal aorta following 1 week AngII treatment. **C**, Representative CPH, SM-actin and DAPI stained abdominal aorta from fl/fl mice following 1 week AngII. **D**, Representative CPH, SM-actin and DAPI stained abdominal aorta from SM22-Δ mice following 1 week AngII.


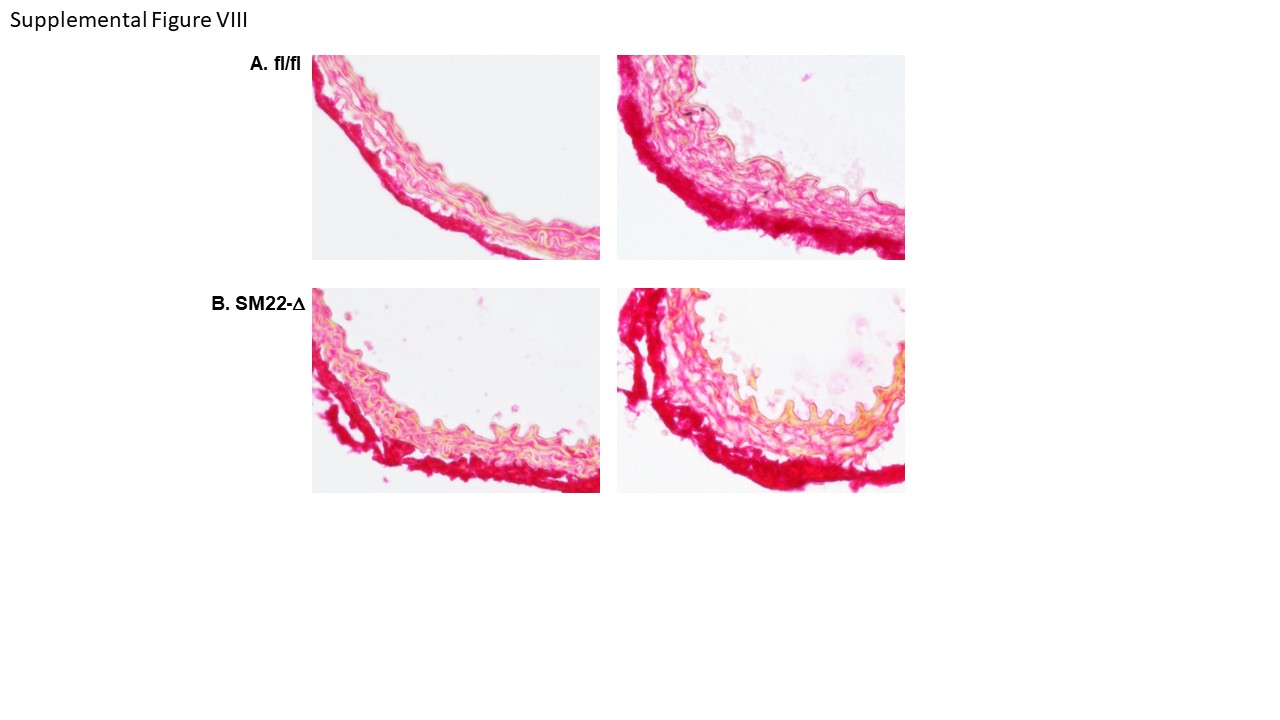


**Supplemental Figure VIII**. Collagen in abdominal aorta tissue. **A**, Representative picrosirius red stained abdominal aorta sections from fl/fl mice following 1 week AngII. **B**, Representative picrosirius red stained abdominal aorta sections from SM22-Δ mice following 1 week AngII.

**
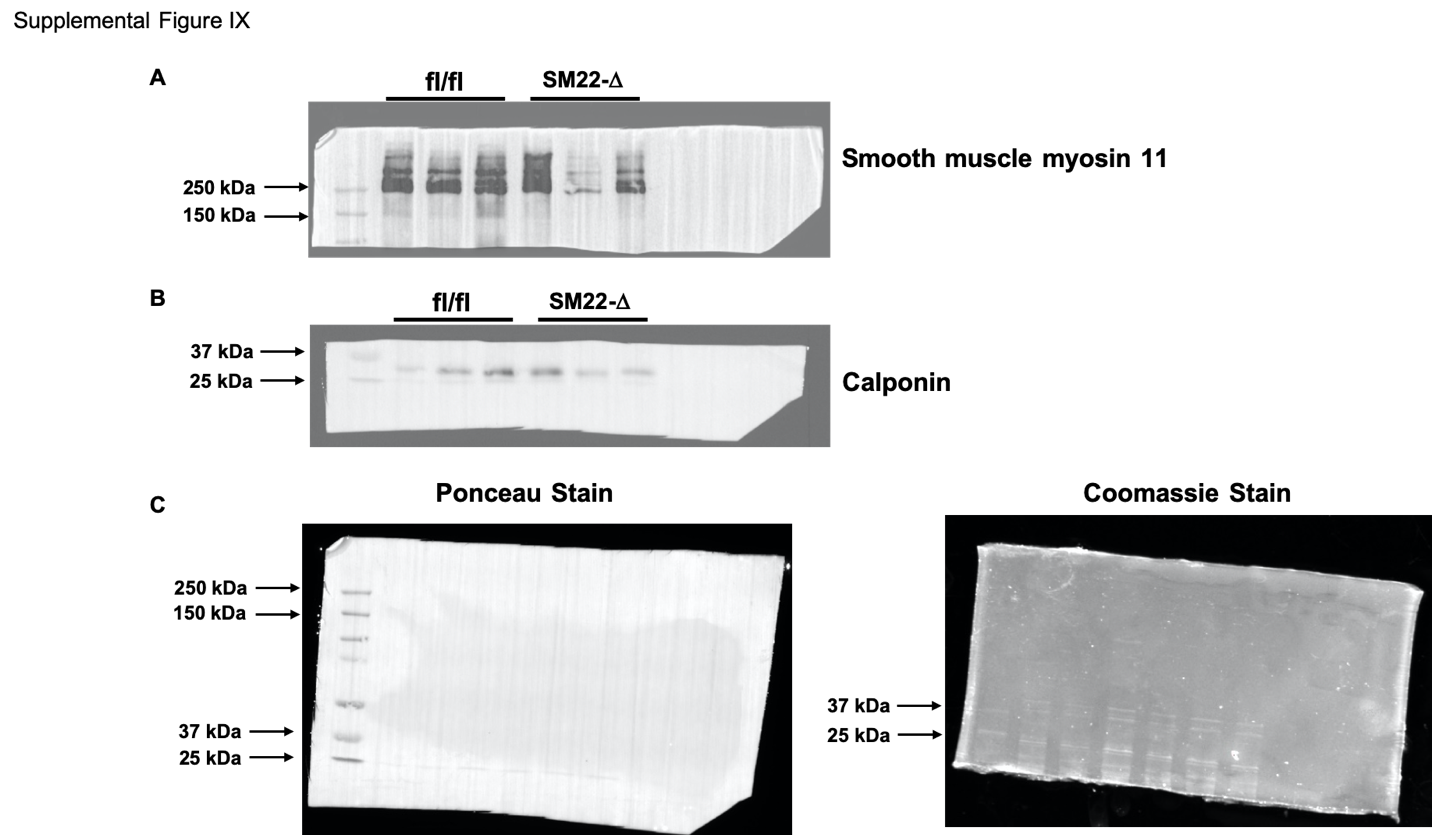
**

**Supplemental Figure IX.** Smooth muscle protein markers in abdominal aortic tissue. **A**, Smooth muscle myosin 11 protein expression in abdominal aortic tissue from fl/fl and SM22-Δ mice following 1 week AngII. **B**, Calponin protein expression in abdominal aortic tissue from fl/fl and SM22-Δ mice following 1 week AngII. **C**, Ponceau stained blot and Coomassie stained gel as loading control.


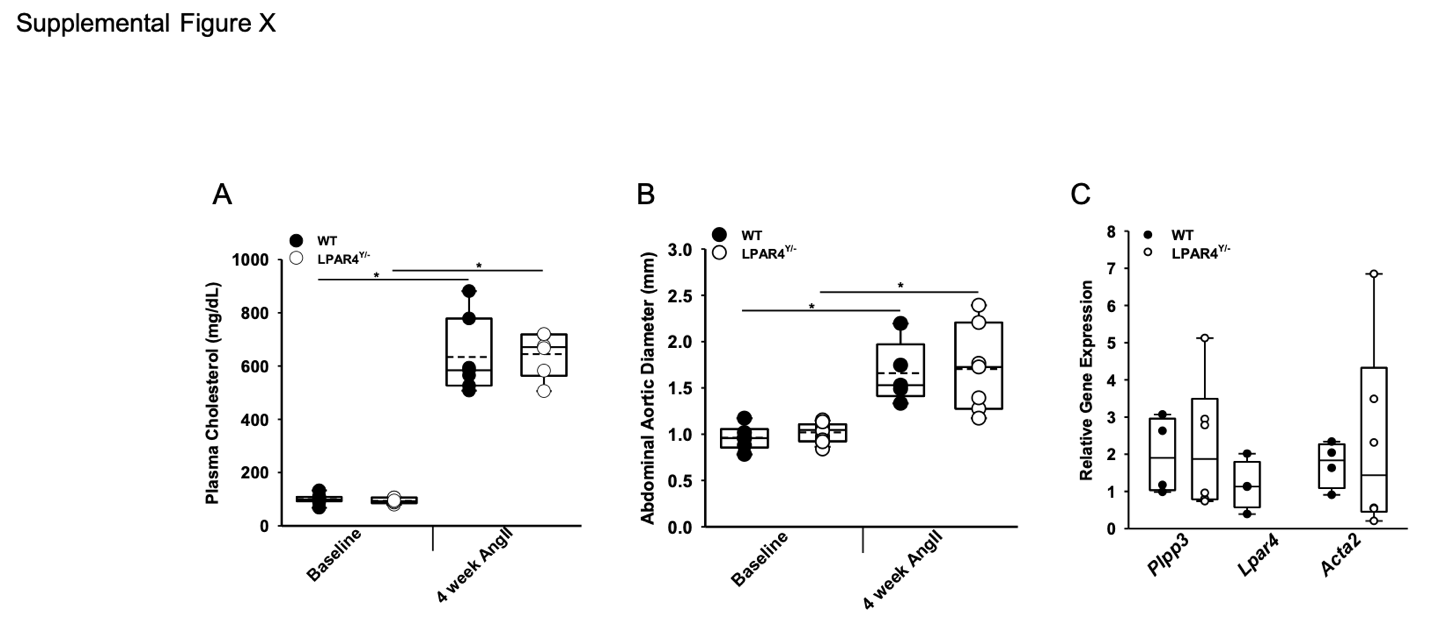


**Supplemental Figure X**. Lysophosphatidic acid receptor 4 knockout mice and abdominal aortic aneurysm. **A**, Plasma cholesterol at baseline and post-4 week AngII were measured in WT (n=9) and *LPAR4Y/-* (n=6) mice, *P<0.001 Two way ANOVA, Bonferroni t-test, all pairwise multiple comparison. **B**, Abdominal aortic diameter measurements of WT (n=6) and *LPAR4Y/-* (n=7) were measured using ultrasound at baseline and 4 week AngII. *P<0.001, Two way ANOVA, Holm-Sidak, all pairwise multiple comparison. **C**, Abdominal aorta gene expression from WT (n=5) and LPAR4Y/- (n=5) mice following 4 week AngII treatment. Data is represented as relative gene expression (WT vs LPAR4^-/-^, following 4 week AngII treatment).


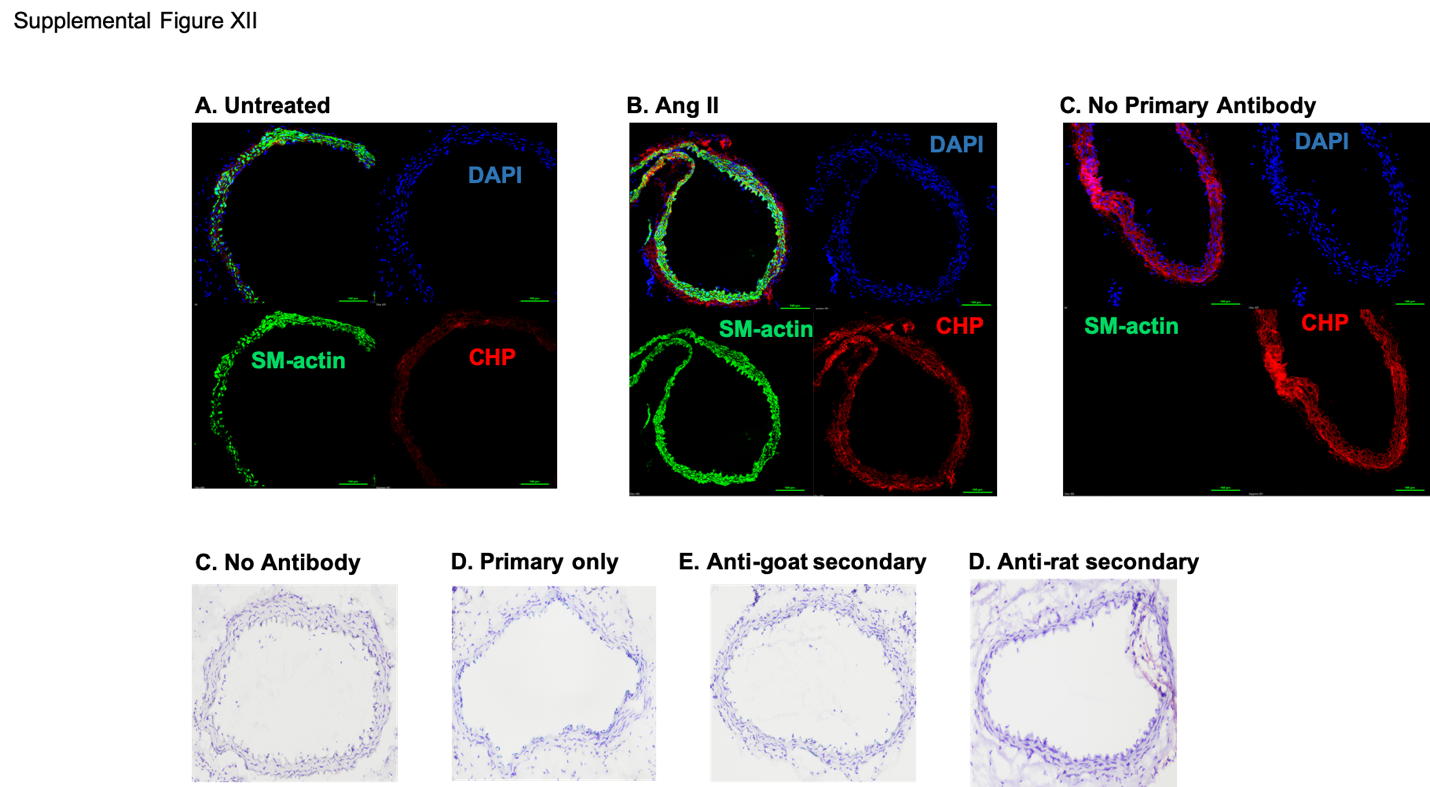


Supplemental Figure XI

**Supplemental Figure XI**. Antibody staining controls. **A**, Untreated abdominal aorta tissue stained with SM-actin, CHP and DAPI. **B**, AngII treated abdominal aorta tissue stained SM-actin, CHP and DAPI. **C**, Abdominal aorta tissue stained with no primary antibody for SM-actin and CHP. **D-G**, Antibody staining controls.
